# 1-Anilino-8-Naphthalene Sulfonate (ANS) Is Not a Desirable Probe for Determining the Molten Globule State of Chymopapain

**DOI:** 10.1371/journal.pone.0050633

**Published:** 2012-11-29

**Authors:** Atiyatul Qadeer, Gulam Rabbani, Nida Zaidi, Ejaz Ahmad, Javed M. Khan, Rizwan H. Khan

**Affiliations:** Interdisciplinary Biotechnology Unit, Aligarh Muslim University, Aligarh, India; Russian Academy of Sciences, Institute for Biological Instrumentation, Russian Federation

## Abstract

The molten globule (MG) state of proteins is widely detected through binding with 1-anilino-8-naphthalene sulphonate (ANS), a fluorescent dye. This strategy is based upon the assumption that when in molten globule state, the exposed hydrophobic clusters of protein are readily bound by the nonpolar anilino-naphthalene moiety of ANS molecules which then produce brilliant fluorescence. In this work, we explored the acid-induced unfolding pathway of chymopapain, a cysteine proteases from *Carica papaya*, by monitoring the conformational changes over a pH range 1.0–7.4 by circular dichroism, intrinsic fluorescence, ANS binding, acrylamide quenching, isothermal titration calorimetry (ITC) and dynamic light scattering (DLS). The spectroscopic measurements showed that although maximum ANS fluorescence intensity was observed at pH 1.0, however protein exhibited ∼80% loss of secondary structure which does not comply with the characteristics of a typical MG-state. In contrast at pH 1.5, chymopapain retains substantial amount of secondary structure, disrupted side chain interactions, increased hydrodynamic radii and nearly 30-fold increase in ANS fluorescence with respect to the native state, indicating that MG-state exists at pH 1.5 and not at pH 1.0. ITC measurements revealed that ANS molecules bound to chymopapain *via* hydrophobic interaction were more at pH 1.5 than at pH 1.0. However, a large number of ANS molecules were also involved in electrostatic interaction with protein at pH 1.0 which, together with hydrophobically interacted molecules, may be responsible for maximum ANS fluorescence. We conclude that maximum ANS-fluorescence alone may not be the criteria for determining the MG of chymopapain. Hence a comprehensive structural analysis of the intermediate is essentially required.

## Introduction

The process of protein folding, despite being one of the most intensely investigated areas, remains obscure in terms of its detailed molecular mechanism. The spontaneity and extreme cooperativity of this process makes it a challenging task to unravel the entire mechanism mainly because of the inability to populate the distinct intermediate states that encompass the folding pathway of a nascent polypeptide [1]–[3]. Characterization of these intermediates is the key to unlock the step by step mechanism and to extend our knowledge on the principles governing protein folding [4], [5].

In past, a lot of attention has been paid to molten globule (MG), an intermediate with feasible occurrence as a general physical state in the folding pathway of globular proteins [6]–[8]. The MG state generally corresponds to late folding intermediate and has been obtained for many proteins under different solvent conditions [9]–[12]. In order to be defined as a typical molten globule, an intermediate requires being a compact collapsed state with substantial amount of secondary structure, loose tertiary contacts without tight side chain packing and a solvent accessible hydrophobic core [13]. Conventionally, the exposed hydrophobic clusters of folding intermediates are detected through 1-anilino-8-naphthalene sulfonate (ANS) binding, a much utilized fluorescence probe for detecting the non-polar character of proteins and membranes [14]. In fact, the available literature reveals that molten globule state is mostly pinpointed from its maximum ANS binding ability under the conditions studied since the other typical features *viz* pronounced secondary structure and disrupted tertiary contacts often seem to merge with intermediates lying in the vicinity of MG-state [12], [15]–[20]. Such utilization of ANS which is based upon the principle that ANS is practically non fluorescent in water but produces brilliant fluorescence upon binding to hydrophobic sites of protein [21] generally ignores the contribution of sulfonate group that was earlier considered as a mere solublizing agent for otherwise almost water-insoluble anilino-naphthalene moiety. However, the role of electrostatic interactions in ANS-protein interaction was highlighted later in the findings that ANS binding to proteins depends upon protein cationic charge and pH of the solution and occurs precisely through ion-pair formation between sulfonate group of ANS and side chains of Arg/Lys/His residues of polypeptide chain [22], [23]. A more recent study has revealed that interaction of sulfonate group of ANS with charged centers of Arg and Lys residues resulted in enhanced fluorescence along with blue shifted emission maxima [24]. Moreover, an increase in ANS fluorescence may not always be associated with formation of MG-state as it may also point to the existence of protein aggregates [25], [26] **.** All these observations compel one to ponder whether maximum ANS-fluorescence always indicates the formation of MG-state?

**Figure 1:**
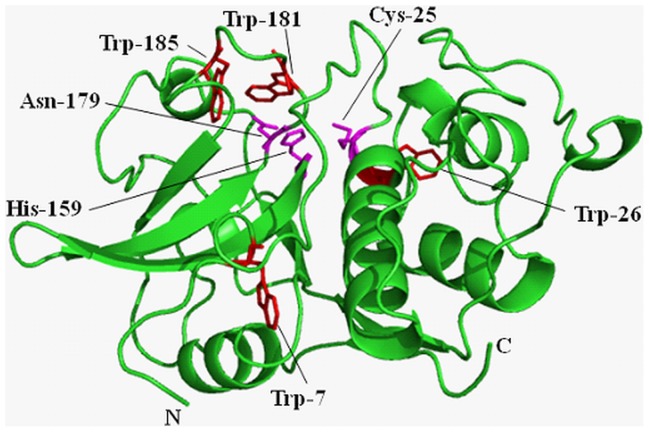
Ribbon structure of chymopapain (1YAL) as drawn by PyMol. Position of Trp and active site residues is shown.

Chymopapain (EC 3.4.22.6), a cysteine protease isolated from latex of *Carica papaya* belongs to α+β class of proteins [27] (Fig. 1) and shares considerable sequence similarity with other cysteine proteases including papain, actinidine, protease Ω and stem bromelain [28]–[30] providing an indication for their polypeptide chains having common folding pattern. Contrary to the wealth of information available on denaturation and refolding aspects of papain and other related proteases [9], [15], [31]–[33], few such reports exist about chymopapain. On the basis of maximum ANS binding capacity, chymopapain has been reported to exist in MG-state at pH 1.0 [34]. Detailed study, however, is required to explore the conformational alterations brought about under acidic conditions. The present study deals with characterization of MG-state of chymopapain as monitored by circular dichroism, intrinsic fluorescence, ANS binding, acrylamide quenching and isothermal titration calorimetry. The hydrodynamic radii of protein at different pH were determined through dynamic light scattering. The study revealed that the although ANS binds maximally to the protein at pH 1.0 as reported earlier, however the secondary structure content of this state is too low for a molten globule. Instead, the conformation of protein examined at pH 1.5 has all the characteristics of a MG-state.

**Figure 2:**
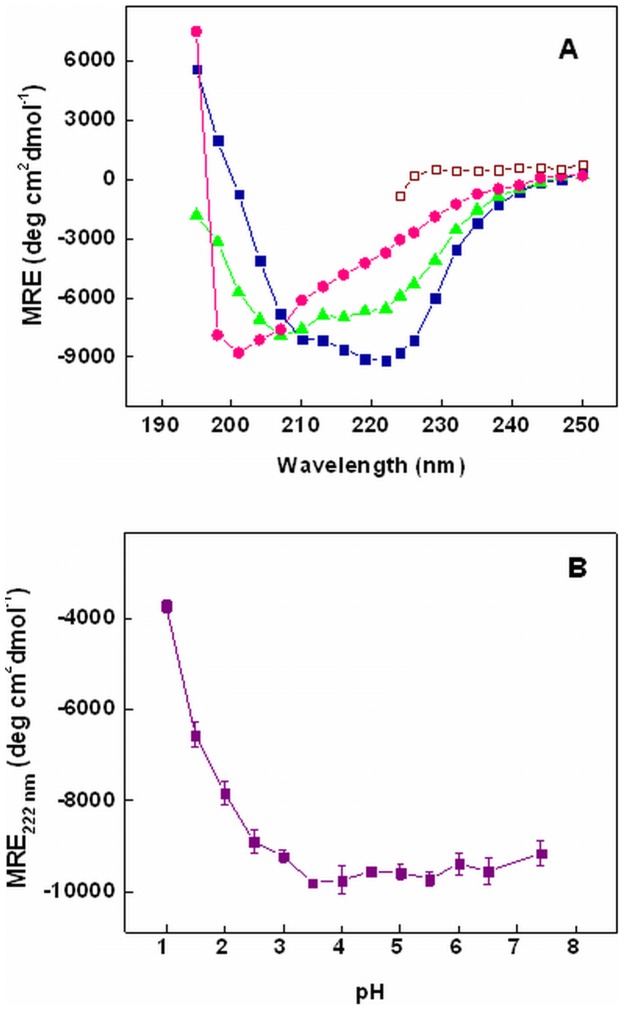
Effect of pH on far-UV CD of chymopapain. (A) Far-UV CD spectra of chymopapain at pH 7.4 (-▪-), pH 1.5 (-▴-), pH 1.0 (-•-) and in the presence of 6M GuHCl (-□-). (B) pH dependent changes in mean residual ellipticity (MRE) at 222 nm of chymopapain. Protein concentration was 8 µM.

## Materials and Methods

Chymopapain from *Carica papaya* (C8526), guanidine hydrochloride (GuHCl) and ANS were purchased from Sigma-Aldrich Chemical Co., St. Louis, MO, USA. All other reagents used were of analytical grade.

**Table 1:** Spectroscopic properties of chymopapain at different pH and under denatured condition.^a^

	Secondary structure			Tertiary structure			ANS binding	
pH	MRE_222 nm_ b	α-helix (%)	[θ]_208_/[θ]_222_	MRE_272 nm_ b	RFI_345 nm_ c	λ_max_ (nm)	RFI_480 nm_ c	λ_max_ (nm)
7.4	−9177.72±228.34	22.6±0.75	0.77±0.03	6549.37±282.43	100±2.91	345±0.23	2.44±1.47	515±0.56
3.0	−9222.14±120.63	22.7±0.40	0.90±0.02	7594.58±433.93	48.5±3.53	339±0.42	6.67±0.38	495±0.42
1.5	−6584.86±163.60	14.0±0.54	1.17±0.02	3602.96±129.83	42.8±2.18	342±0.14	79.13±5.55	483±0.56
1.0	−3725.50±162.74	4.6±0.54	1.94±0.02	−95.57±20.38	38.4±0.56	344±0.23	100.00±5.24	481±0.28
6M GuHCl	−765.03±80.59	–	–	−8.91±1.89	78.8±4.21	351±0.50	5.11±0.90	515±1.10

a The data are average with standard deviation of three independant trials.

b MRE, deg cm^2^ dmol^−1^

c Relative fluorescence intensity.

### Sample Preparation

A stock solution of protein was prepared in 20 mM sodium phosphate buffer pH 7.4 and was dialyzed at 4°C. Protein concentration was determined by using 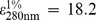 [35], on Perkin Elmer (Lambda 25) double beam spectrophotometer. The autocatalysis was checked by trichloroacetic acid (TCA) precipitation method [36]. Acid denaturation of chymopapain was carried out in the pH range 1.0–7.4. pH measurements were carried out on Mettler Toledo pH meter (Seven Easy S20–K) using Expert “Pro3 in 1” type electrode. The least count of the pH meter was 0.01 pH unit. The protein samples for acid denaturation studies were prepared in 20 mM of following buffers: KCl-HCl (pH 1.0–1.5), Gly-HCl (pH 2.0–3.0), sodium acetate (pH 3.5–5.5), sodium phosphate (pH 6.0–7.4). All the buffers were filtered through PVDF 0.45 µm syringe filter (Millipore Millex-HV). Before spectrophotometric measurements, chymopapain was allowed to incubate in buffer of desired pH for 1 hr at room temperature. The ANS concentration was determined spectrophotometrically using 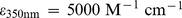. The GuHCl concentration was determined by refractive index measurement.

**Figure 3:**
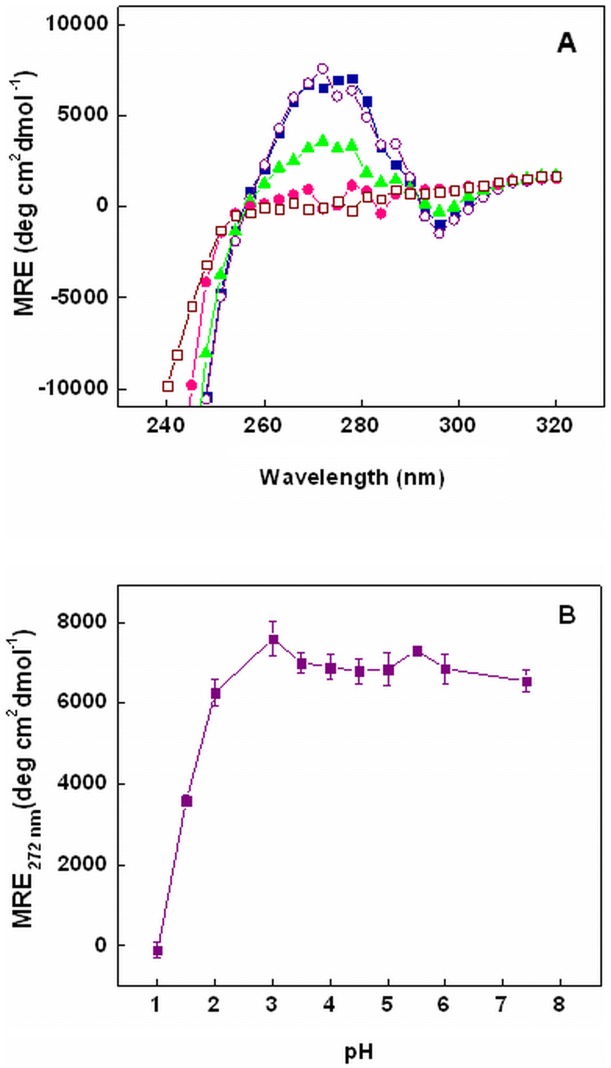
Effect of pH on near-UV CD of chymopapain. (A) Near-UV CD spectra of chymopapain at pH 7.4 (-▪-), pH 3.0 (-○-), pH 1.5 (-▴-), pH 1.0 (-•-) and in the presence of 6M GuHCl (-□-). (B) pH dependent alterations in mean residual ellipticity (MRE) at 272 nm of chymopapain. Protein concentration was 40 µM.

### Circular Dichroism

CD measurements were carried out on Jasco spectropolarimeter (J–815) equipped with a Jasco Peltier-type temperature controller (PTC–424S/15). The instrument was calibrated with d-10-camphorsulphonic acid. All the CD measurements were carried out at 25°C with scan speed of 100 nm/min and response time of 1 s. Protein concentration for far-UV and near-UV measurements were 8 and 40 µM respectively. The pathlength of cells were 1 mm for far-UV and 10 mm for near-UV CD. Each spectrum was the average of 2 scans. The results were expressed as MRE (Mean Residue Ellipticity) in deg cm^2^ dmol^−1^ which is defined as:
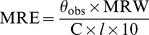
(1)where θ_obs_ is the CD in milli-degree, MRW is the mean residue weight (110), *l* is the path length of the cell in cm and C is concentration in mg/ml. Helical content was calculated from the MRE values at 222 nm using the following equation as described by Chen *et al.* [37]:
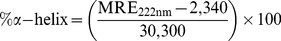
(2)


**Figure 4:**
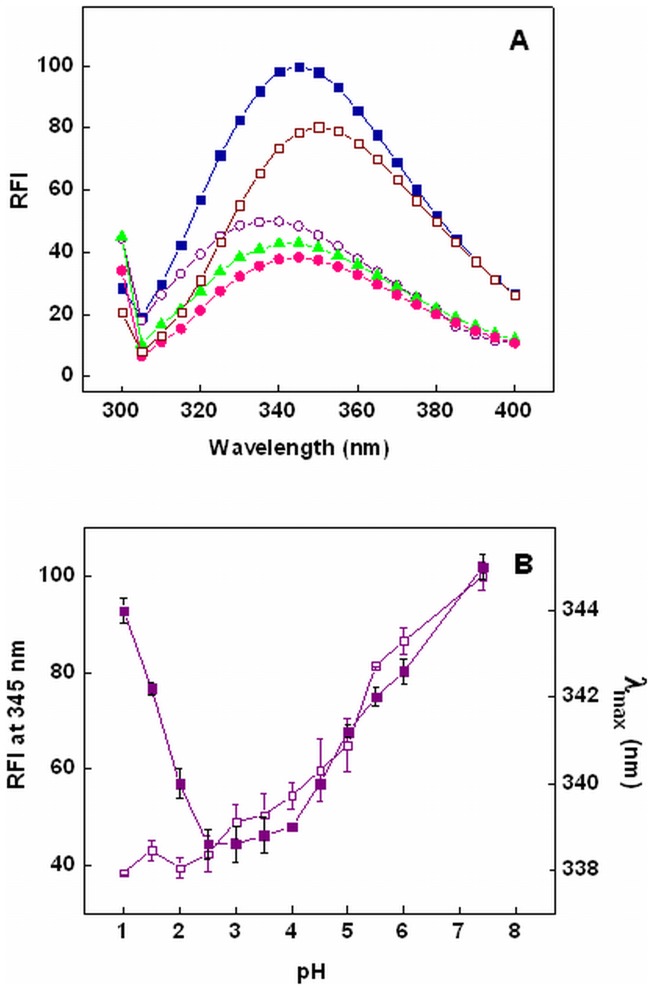
pH dependence of Trp fluorescence of chymopapain. (A) Fluorescence emission spectra of chymopapain at pH 7.4 (-▪-), pH 3.0 (-○-), pH 1.5 (-▴-), pH 1.0 (-•-) and in the presence of 6M GuHCl (-□-). (B) Changes in relative fluorescence intensity (RFI) at 345 nm (-□-) and emission maxima (-▪-) of chymopapain as a function of pH. Spectra were obtained by exciting Trp at 295 nm. Protein concentration was 8 µM.

### Intrinsic and Extrinsic Fluorescence

The steady-state fluorescence measurements were performed on Hitachi spectrofluorimeter (F–4500). The fluorescence spectra were measured at 25°C. Both excitation and emission slits were set at 5 nm. The protein samples (8 µM) were excited at 295 nm and emission spectra were recorded from 300 to 400 nm. For ANS binding experiment, protein samples at different pH were incubated with 50 fold molar excess of ANS for 30 min at 25°C in dark. The excitation wavelength for ANS fluorescence was set at 380 nm and the emission spectra were recorded from 400 to 600 nm.

**Figure 5:**
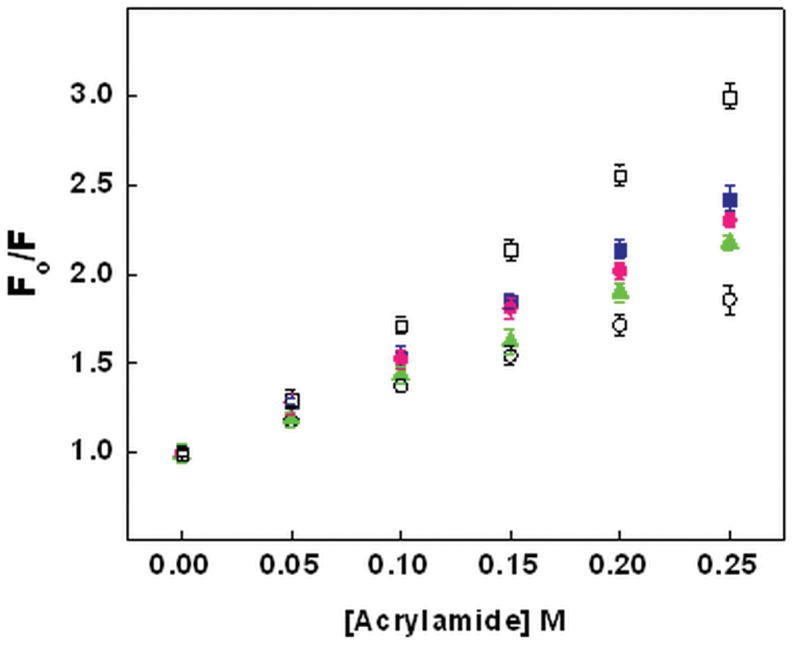
Stern-Volmer plot obtained from acrylamide quenching of chymopapain. Plot at pH 7.4 (-▪-), pH 3.0 (-○-), pH 1.5 (-▴-), pH 1.0 (-•-) and in the presence of 6M GuHCl (-□-). Protein concentration was 8 µM.

**Table 2:** Acrylamide quenching data of chymopapain at different pH and under denatured condition.^a^

pH	*K*_sv_ (M^−1^)	*K*_q_×10^8^ (M^−1^s^−1^)	*R^2^*
7.4	5.59±0.06	13.0±0.14	0.9988±0.001
3.0	3.40±0.06	7.9±0.13	0.9974±0.001
1.5	4.58±0.05	10.6±0.11	0.9830±0.001
1.0	5.21±0.03	12.1±0.07	0.9985±0.001
6M GuHCl	8.02±0.07	18.6±0.15	0.9986±0.001

a The data are average with standard deviation of three independent trials.

**Figure 6:**
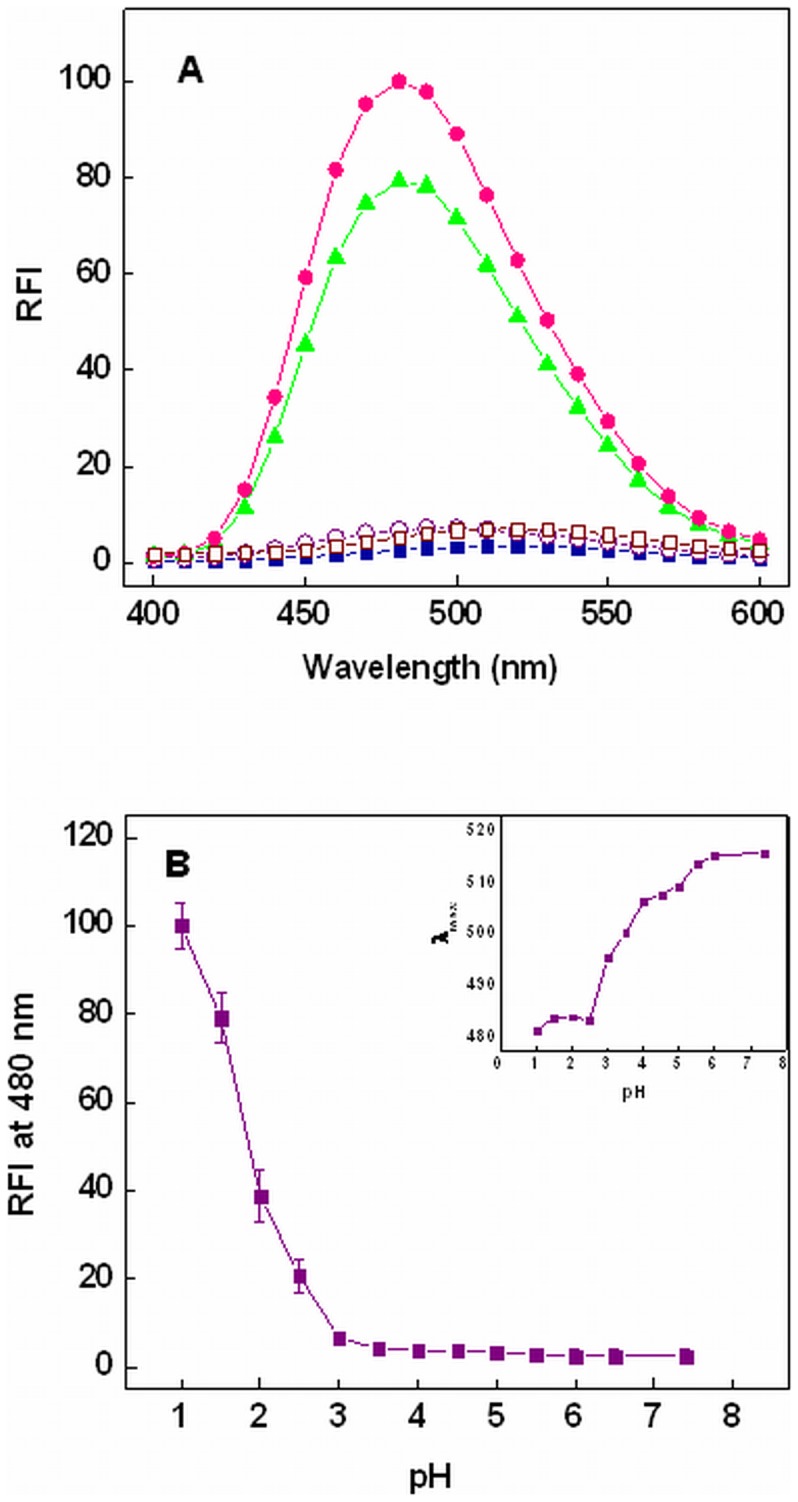
Effect of pH on ANS binding of chymopapain. (A) ANS fluorescence spectra of chymopapain at pH 7.4 (-▪-), pH 3.0 (-○-), pH 1.5 (-▴-), pH 1.0 (-•-) and in the presence of 6M GuHCl (-□-). (B) Changes in ANS fluorescence intensity of chymopapain as a function of pH at 480 nm. Inset: pH dependence of ANS emission maxima (λ_max_) of chymopapain. Protein concentration was 8 µM. The molar ratio of protein to ANS was 1∶50.

### Acrylamide Quenching

Fluorescence quenching experiments were performed by adding aliquots from stock solution of 5 M acrylamide (quencher) to protein solutions (8 µM) in order to achieve the desired range of quencher concentration. Samples were excited at 295 nm and the emission spectrum was recorded from 300 to 400 nm. The decrease in fluorescence intensity of protein at emission maxima was analyzed by using the Stern–Volmer equation:
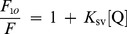
(3)where *F*_o_ and *F* are the fluorescence intensities in the absence and presence of quencher respectively, *K*_sv_ is the Stern-Volmer quenching constant which was calculated as the slope of *F*_o_/*F vs.* [Q] plot and [Q] represents the molar concentration of quencher. The bimolecular rate constant *K*_q_ was calculated from the relation:
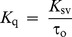
(4)where τo is the mean fluorescence life time of Trp which is ∼4.31×10−9 s [38].

### Isothermal Titration Calorimetry

The ITC measurements were performed on VP-ITC titration calorimeter from Microcal (Northampton, MA). The samples were thoroughly degassed under vacuum in the Thermovac unit supplied with the instrument. All the experiments were performed at 25°C. The sample cell was filled with 25 µM chymopapain incubated at desired pH and the reference cell contained respective buffer. The titrations were carried out using ANS solutions prepared in desired buffers. Duration of each injection was 20 s and the time delay to allow equilibration between successive injections was 180 s. Stirring speed was 307 rpm and reference power was 16 µcal/s. Control experiments were performed to correct the data for the heats of dilution of ligand and buffer mixing. The heat signals obtained from ITC were integrated using Origin 7.0 software supplied by Micro Cal Inc. The binding of ANS to the acid-induced states of chymopapain fitted best to two sets of independent binding sites. This binding model defines association constants as:
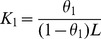
(5)

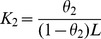
(6)where θ is the fraction of sites occupied by the ligand. The total heat content of the solution is given by:
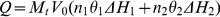
(7)where Mt is the total concentration of the macromolecule, V0 is the active cell volume, n1 and n2 represent the number of ligand molecules bound to first and second set of sites respectively, ΔH1 and ΔH2 correspond to the enthalpy change associated with respective binding sites. The heat released ΔQ(i) from the ith injection for an injection volume dVi is given by:
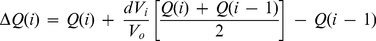
(8)which is then used in the Marquardt minimization algorithm to obtain best fitting values until constant χ2 values were achieved [39], [40].

**Figure 7:**
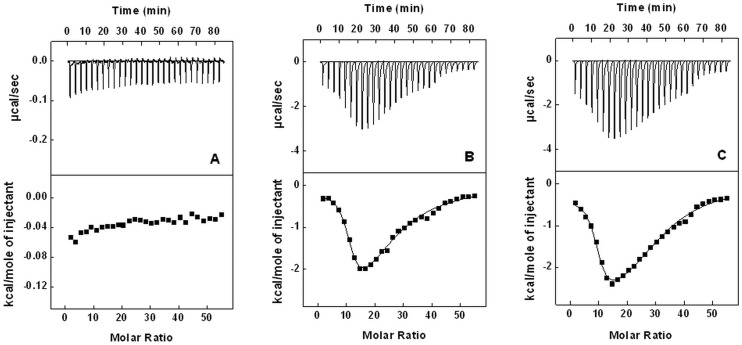
Isothermal titration calorimetery. Plot of enthalpy change against molar ratio for the titration of ANS with chymopapain at (A) pH 7.4 (B) pH 1.5 and (C) pH 1.0. The upper panels represent the raw data and the bottom panels are the non-linear best fit data. Protein concentration was 25 µM.

**Table 3:** Number of moles and thermodynamic parameters for binding of 1-anilino-8-naphthalene sulfonate (ANS) to chymopapain at different pH.

	High affinity binding site				Low affinity binding site			
pH	N_1_	K_1_ (M^−1^)	ΔH_1_ a	TΔS_1_ a	N_2_	K_2_ (M^−1^)	ΔH_2_ a	TΔS_2_ a
1.0	8.9±0.2	(5.1±1.7)×10^5^	−0.3±0.0	7.4	22.1±1.1	(7.8±1.0) ×10^3^	−3.4±0.2	1.0
1.5	10.5±0.3	(3.4±1.3)×10^5^	−0.2±0.0	7.3	15.2±2.0	(5.6±1.0) ×10^3^	−4.0±0.6	1.1

a (kcal mol^−1^).

### Dynamic Light Scattering

DLS measurements were carried out with a protein concentration of 40 µM in buffers of desired pH. The experiment was performed at 830 nm using DynaPro–TC–04 dynamic light scattering equipment (Protein Solutions, Wyatt Technology, Santa Barbara, CA) equipped with a temperature-controlled microsampler. Prior to measurements, the samples were centrifuged at 10,000 rpm for 10 min and were filtered through 0.22 µm pore sized microfilter (Whatman International, Maidstone, UK) directly into a 12 µl black quartz cell while ensuring removal of any trapped air bubble. Measured size was presented as an average of 20 scans taken at 25°C. The data were analysed by Dynamics 6.10.0.10 software at optimized resolution. The mean hydrodynamic radius (*R*_h_) and polydispersity (*P*_d_) was estimated on the basis of an autocorrelation analysis of scattered light intensity data based on translational diffusion coefficient by Stokes-Einstein equation:
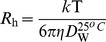
(9)where *R*_h_ is the hydrodynamic radius, *k* is the Boltzmann constant, T is the absolute temperature, *η* is the viscosity of water and 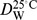 is the translational diffusion coefficient.

**Figure 8:**
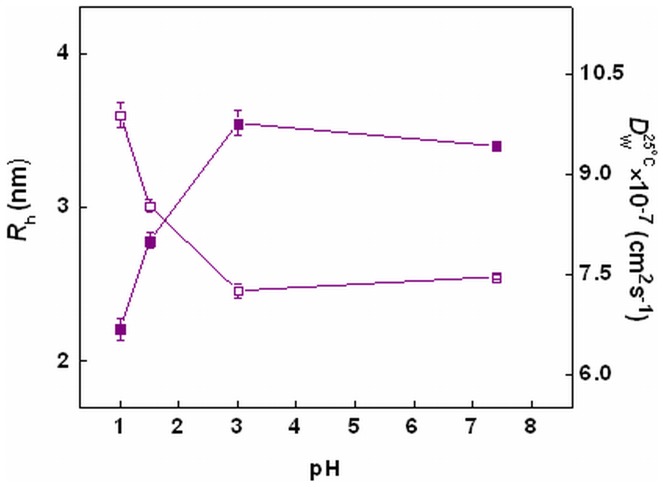
Dynamic light scattering. Effect of pH on hydrodynamic radii (-□-) and translational diffusion coefficient (-▪-) of chymopapain. Protein concentration was 40 µM.

**Table 4:** Comparison of hydrodynamic radii (*R*_h_) and translational diffusion coefficients (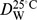) of chymopapain at different pH.^a^

pH	*App. M.W.* (kDa)	*R*_h_ (nm)	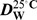 ×10^−7^ (cm^2^s^−1^)	*P_d_* (%)
7.4	30.1±0.47	2.6±0.02	9.42±0.06	8.4±0.64
3.0	27.9±1.20	2.5±0.05	9.76±0.19	16.3±0.02
1.5	44.4±1.45	3.0±0.04	8.00±0.11	9.0±0.93
1.0	67.6±3.59	3.6±0.08	6.68±0.16	11.7±0.46
6 M GuHCl	169.5±8.94	5.1±0.12	3.99±0.10	22.1±8.29

a The data are the average with standard deviation of three independent trials.

## Results

### Changes in Secondary Structure Monitored by Far-UV CD

The differential absorption of a protein molecule in far-UV region (190–250 nm) arises due mainly to peptide bonds which undergo a weak but broad n→π* transition centered around 220 nm and a sharp π→π* transition near 190 nm [41]. Thus CD spectra of a protein in this region can be exploited to monitor the changes in secondary structure. A comparison of far-UV CD spectra of chymopapain obtained at different pH is shown in Fig. 2A. Protein in native state (pH 7.4) exhibited minima at 208 and 222 nm, the latter being more prominent, indicating the presence of α-helical structure. Most of the spectral features of native state were retained at pH 1.5 suggesting the presence of substantial amount of secondary structure. This is also evident from the calculated % α-helical content of protein (Table 1). However at pH 1.0, the minima at 208 and 222 nm were reduced to a single negative peak around 200 nm indicating significant loss of secondary structure and acquisition of random coil like conformation [41]. The denatured state of protein (6M GuHCl) appeared to have lost all elements of secondary structure. To explore the influence of acidic pH on secondary structure of chymopapain, we followed changes in mean residual ellipticity (MRE) at 222 nm (Fig. 2B). Apparently no changes in the ellipticity values were observed in the pH range 3.0–7.4 though some structure induction was observed at pH 3.0 where MRE_222 nm_ was slightly greater than the native state [Table 1]. A gradual but invariable decrease in ellipticity was noticed below pH 3.0 suggesting that enzyme continuously lost its secondary structure. In comparison to native state of protein at pH 7.4 where the MRE_222 nm_ was considered as 100%, the ellipticity was reduced to 72% at pH 1.5 and to mere 40% at pH 1.0 indicating that secondary structure is largely maintained at pH 1.5 but was considerably lost under latter condition. Table 1 summarizes all the spectroscopic features of chymopapain at different pH as well as under denatured condition.

**Figure 9:**
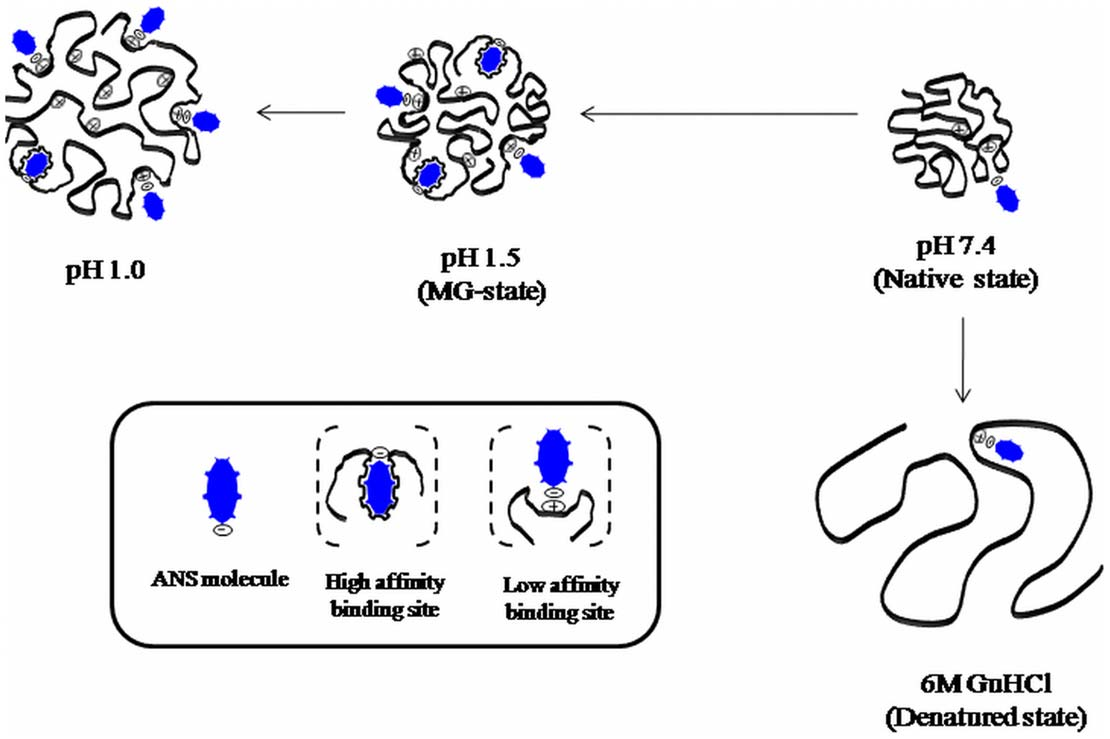
Schematic representation of pH-induced unfolding pathway of chymopapain. The probable mode of binding of ANS molecules at different pH as well as under denatured condition is shown.

### Changes in Tertiary Structure Monitored by Near-UV CD

The response of tertiary structure of chymopapain against pH change was followed by near-UV CD measurements. Fig. 3A and 3B show near-UV CD spectra and trend in MRE at 272 nm respectively of chymopapain at different condition. Native state of protein revealed a broad maxima around 268–277 nm arising from Phe and Tyr side chains and a trough at 295 nm contributed by Trp residues. With slight changes, the ordered conformation of native state remained similar to as low as pH 3.0. An increase in the MRE_272 nm_ at pH 3.0 with respect to the native state [Table 1] may indicate formation of additional tertiary contacts as a consequence of structure induction as noticed in far-UV CD results. The signals were diminished at pH 1.5 indicating disruption of asymmetrical environment around aromatic chromophore, thus a loss of rigid tertiary structure. The tertiary contacts were significantly abolished at pH 1.0 and the spectra resemble that of 6M GuHCl denatured state.

### Tertiary Conformational Alterations Probed by Tryptophan Fluorescence

Intrinsic fluorescence parameters such as fluorescence intensity (FI) and emission maxima (λ_max_) provide a very sensitive mean for studying structural dynamics and polarity of protein chromophore *viz* Trp, Tyr and Phe. The pH dependant changes in intrinsic fluorescence of chymopapain were measured by specifically exciting Trp at 295 nm and following conformational alterations in its vicinity. Chymopapain has four Trp residues, three out of which are located in R-domain and one in L-domain [27] (Fig. 1). As shown in Fig. 4A, native state of chymopapain exhibited emission maxima at 345 nm which suggests that Trp residues are largely exposed to the solvent, an observation consistent with the crystal structure of enzyme. At pH 3.0, FI was reduced to 50% along with a blue shift of 6 nm indicating that Trp microenvironment became non-polar. Probably the structure induction (as observed in CD results) has fetched some of the Trp residues in a non-polar environment resulting in formation of a compact state at pH 3.0. Progressive lowering of pH led to rise in λ_max_ with further decrease in FI. The λ_max_ for 6M GuHCl denatured state was red shifted to 351 nm with concomitant decrease in FI indicating that Trp residues are maximally exposed to the solvent. Fig. 4B summarizes pH dependant changes in FI and λ_max_ of chymopapain. A significant decline in FI and λ_max_ was noticed between pH 7.4 and pH 3.0. As pH was lowered further the emission maxima began to increase, crossing a value of 342 nm at pH 1.5 and finally reaching 344 nm at pH 1.0 with simultaneous decrease in FI. These observations suggest that protein conformation under acidic conditions is different from native and 6M GuHCl denatured state.

### Evaluation of Trp Accessibility by Acrylamide Quenching

Fluorescence quenching by acrylamide was carried out to determine the extent of exposure of Trp residues under different pH conditions and hence to derive information about conformational changes in protein. Fig. 5 depicts the Stern-Volmer plot for chymopapain in different states while Table 2 describes the respective values of Stern-Volmer constant (*K*_sv_). As can be observed, *K*_sv_ for protein at different pH followed the order 5.59 M^−1^ (pH 7.4), 5.21 M^−1^ (pH 1.0), 4.58 M^−1^ (pH 1.5) and 3.4 M^−1^ (pH 3.0). The *K*_sv_ for 6M GuHCl denatured chymopapain was highest among all states (8.02 M^−1^). Additionally, the bimolecular rate constant (*K*_q_) was calculated at different pH in order to confirm the static or dynamic type of quenching. Table 2 reveals that *K*_q_ value in all the cases were of the order of 10^8 ^M^−1^s^−1^ which is almost 100 times lower than the maximum permissible value for dynamic quenching i.e. 2×10^10^ M^−1 ^s^−1^ [42].

### Detection of Exposed Hydrophobic Clusters by ANS Binding

The folding intermediates of globular protein are frequently distinguished from their native or denatured state through change in ANS fluorescence. In a well folded (native state) or fully unfolded (denatured state) protein, the hydrophobic patches are unavailable for binding with ANS molecules as they are buried in native state while significantly disrupted when protein is denatured. The partial unfolding of the protein molecule exposes these hydrophobic molecules which can interact with ANS molecules producing an enhanced ANS fluorescence as well as blue shifted emission maxima [43]. A comparison of ANS fluorescence spectra of chymopapain at different pH is shown in Fig. 6A. As expected, the spectra of native and 6M GuHCl denatured state showed negligible ANS fluorescence. Also, ANS fluorescence was insignificant at pH 3.0 where protein seems to have acquired a compact conformation which did not allow access of ANS molecules to the buried hydrophobic patches of protein. At pH 1.5, ANS-FI was ∼30 times more than native state indicating enhanced exposure of hydrophobic patches (Table 1) while maximum FI was observed following incubation at pH 1.0. Besides, binding of ANS to the protein resulted in a prominent blue shift in λ_max_ at low pH. A plot of ANS-FI at 480 nm of chymopapain as a function of pH is shown in Fig. 6B. Apparently no change in FI was observed from pH 7.4 to pH 3.0. The increase in FI was distinguishable below pH 3.0 and attained maximum value at pH 1.0. Protein in native state exhibited maximum emission of ANS fluorescence at 515 nm which decreased to 481 nm at pH 1.0 (Fig. 6B inset). The maximum FI as well as remarkable blue shift may prompt one to conclude that molten globule state of chymopapain probably exists at pH 1.0. However, far-UV CD data was quite inconsistent with this presumption as protein was found to retain very less secondary structure at pH 1.0. Therefore further experiments were required to trace the actual MG-state.

### Determining the Interactive Forces between ANS and Chymopapain by ITC

ITC measurements were carried out in order to examine the type of interactions between ANS and chymopapain at pH 7.4 (Fig. 7A), pH 1.5 (Fig. 7B) and pH 1.0 (Fig. 7C). The upper panel in the figures represents the thermogram of raw signals obtained from the titration of ANS with chymopapain where each peak represents a single injection of ANS solution. The lower panel shows the amount of heat liberated per injection as a function of molar ratio of ANS to protein. The ANS binding isotherm of chymopapain obtained at pH 7.4 could not be fitted to any binding model because binding of ANS with protein did not generate an appreciable amount of heat. The binding isotherms generated at pH 1.5 and pH 1.0 show best non-linear fitting to two independent binding sites with each site binding to more than one molecule of ANS. The thermodynamic parameters obtained from model fitting are summarized in Table 3. As can be seen, at both pH 1.5 and 1.0 the binding of ANS molecules to the protein at high affinity binding site (K_1_ ∼ 10^5^ M**^−^** ^1^) was derived exclusively by a large change in entropy (7.3 and 7.4 kcal mol**^−^** ^1^) as the enthalpic contribution to the binding process was insignificant i.e −0.2 and −0.3 kcal mol^−1^ which is nearly equal to zero. On the other hand, the ANS binding to low affinity binding site (K_2_∼10^3^ M**^−^** ^1^) occurred at the expense of noticeable change in enthalpy (−4.0 and −3.4 kcal mol^−1^) as well as entropy (1.1 and 1.0 kcal mol**^−^** ^1^). It is notable that number of ANS molecules bound to protein at high affinity binding site (n_1_) is more at pH 1.5 than at pH 1.0. Whereas those bound to the low affinity binding site (n_2_) is greater at pH 1.0 than at pH 1.5.

### Changes in Hydrodynamic Radii Examined by DLS

Changes in hydrodynamic radii (*R*_h_) of protein as a function of pH were evaluated through dynamic light scattering measurements (Fig. 8). The *R*_h_ at pH 7.4 was found to be 2.6 nm. A slight decrease in *R*_h_ at pH 3.0 (2.5 nm) may be attributed to the formation of compact state as observed in CD and Trp fluorescence results. Moving further down to pH 1.5 and to pH 1.0 the *R*_h_ increased invariably to 3.0 and 3.6 nm respectively, thus pointing towards continuous unfolding of the molecule driven by structural loss upon extreme acidification. The *R*_h_ was increased by 15.3% at pH 1.5 and 38.4% at pH 1.0 with respect to the native state. Maximum *R*_h_ (5.1 nm) was observed for 6M GuHCl denatured state indicating existence of a remarkably unfolded and expanded conformation. Similar pattern was observed in apparent molecular weight for pH-induced unfolding of chymopapain (Table 4). Since hydrodynamic radii and translational diffusion coefficient (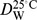), which describes the nature of molecules in solution phase, are inversely related to each other. Therefore the values for 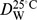 followed opposite pattern with respect to *R*_h_ (Fig. 8 & Table 4). A low value of polydispersity (9.0–22.1%) suggests the existence of almost homogenous species and that protein behaved as monomer under all the conditions studied [16].

## Discussion

Under extreme acidic or alkaline conditions, complete protein denaturation usually remains unaccomplished. This fact, however, is advantageous in a sense that it may often lead to the formation of partially folded states commonly referred to as molten globule. In past, molten globule and related states have gained a lot of attention as they appear to resemble kinetic folding intermediates. Hence their study provides useful clues to several intricate aspects related to protein folding phenomena. pH denaturation studies of chymopapain, a cysteine protease, were thus conducted in an attempt to move a step ahead in this field. The denaturation of protein was carried out over a pH range 1.0–7.4 and the conformational perturbations were monitored through various spectroscopic, calorimetric and hydrodynamic techniques.

Far-UV CD spectra of native state of chymopapain showed negative peak at 208 nm and 222 nm, a sign for the presence of helical structure. When exposed to acidic pH, the protein resisted unfolding up to pH 3.0 indicating considerable structural stability against moderate pH change. However when pH was lowered further, the molecule began to lose its secondary structure continuously without undergoing any reformation even at pH 1.0. Such increase in structural lability at extremely low pH could have been caused by charge-charge repulsion due to the protonation of ionizable side chain [44]. At pH 1.5, chymopapain was found to maintain ∼62% of α-helical structure with respect to native state, while at pH 1.0 the protein acquired a random coil like confirmation with only 20% of the structure retained. The spectra of chymopapain at pH 1.5 was characterized by decreased MRE_222 nm_ but almost unaltered MRE_208 nm_ with respect to the native state. This observation is consistent with that previously reported for the MG-state of β-lactamse and phosphoglycerate kinase [45]. On the other hand, pH 1.0 spectra was similar to the protein with random coil conformation as confirmed from appearance of a single negative peak at 200 nm which is clearly absent in all other conditions. We also calculated the ratio [θ]_208_/[θ]_222_, which probably relies upon structural element packing, at different pH conditions. The values obtained were 0.79, 1.17 and 1.94 for protein incubated at pH 7.4, pH 1.5 and pH 1.0 respectively (Table 1). The ratio 1.17 acquired at pH 1.5 lies well within the range (1.1–1.4) set on the basis of the values observed for the MG-state of a large number of different proteins [45] while the ratio 1.94 obtained at pH 1.0 lies far beyond this range. The data so far indicates that protein exists in MG state at pH 1.5 while exhibits unfolded conformation at pH 1.0. The lack of any extremum in the obtained MRE value at low pH suggests that chymopapain directly transforms into MG-state without undergoing an initial unfolding (under moderate acidic conditions) followed by refolding into a compact molten globule like state (under extreme acidic pH). Both such behaviors (i.e. direct and indirect transformation into MG-state) shown by several different proteins have been reported earlier [44].

Near-UV CD analysis showed that tertiary structure of protein suffered no detectable loss between pH 7.4 and pH 3.0. However at pH 1.5, the tertiary contacts seemed to be significantly disrupted providing further evidence in support of MG-state at this pH. When monitored by intrinsic fluorescence measurements, changes in tertiary structure followed a biphasic transition. The first transition phase from pH 7.4 got stabilized at pH 3.0 where a blue shift of 6 nm (λ_max_ at 339 nm) along with 50% reduction in FI was observed. A blue shifted emission maxima accompanied by decrease in FI was also observed for caricain, another cysteine protease, in the same pH range [34]. The observed changes could be attributed to the fact that Trp-181 and Trp-185 are located in close vicinity of active site residues Cys-25, His-159 and Asn-179 (Fig. 1) and the protonated form of His act as an intrinsic fluorescence quencher [46]. The second transition consisted of a gradual decrease in FI along with an increase in emission maxima to 342 nm at pH 1.5 and finally to 344 nm at pH 1.0. These results indicate that Trp residues initially got buried in non-polar environment with the appearance of a compact state at about pH 3.0, but the structural loss under highly acidic conditions (as observed in far-UV and near-UV CD measurements) led to their exposure again. The 6M GuHCl denatured state exhibited emission maxima at 351 nm, though FI was not as quenched as under acidic conditions. It is well known that response of Trp fluorescence during the unfolding of polypeptide varies from protein to protein depending upon the location of Trp residues. Besides, the fluorescence intensity is quenched not only by the solvent molecules but also by intrinsic quenchers such as disulfide, an amide or an electron accepting group on a neighboring side chain [47]. These facts suggest that, at least for chymopapain, the intrinsic fluorescence quenchers (such as protonated His residue) are probably better able to quench Trp fluorescence than the solvent molecules. Nevertheless, the significant red shift (345 to 351 nm) is an indication that Trp residues of chymopapain are maximally exposed to solvent and the polypeptide indeed exists in denatured state. It is worth mentioning that the Trp FI of acid-induced MG-state of papain was also significantly reduced (45%) along with a 2 nm blue shift unlike its 6M GuHCl denatured state where FI was not much affected but a prominent red shift was observed [31].

The dynamics of a protein molecule in solution is also manifested in the extent of penetration by a quencher molecule through the core of that protein. Thus, quenching constant was determined for different conformational states in order to compare the degree of exposure of aromatic residues. The data was in good agreement with intrinsic fluorescence results. *K*_sv_ was lowest for protein following incubation at pH 3.0 (3.4 M^−1^) further supporting the formation of compact conformation. Due to the shift of Trp residues to the interior of the protein, it might have become less accessible to the solvent and hence to the quencher. With increasing Trp accessibility under extreme acidification, the *K*_sv_ also increased to 4.58 M^−1^ at pH 1.5 and to 5.21 M^−1^ at pH 1.0. *K*_sv_ was maximum for 6M GuHCl denatured state suggesting that Trp residues were maximally exposed to the solvent. Besides, *K*_q_ values confirmed that acrylamide was dynamically quenching the fluorophores in all the samples.

The extrinsic fluorescence results showed that ANS-FI was minimal between pH 3.0 and pH 7.4 demonstrating poor availability of binding sites due to intact molecular structure. At pH 1.5, a marked increase (∼30 times) in ANS fluorescence along with a blue shift of 23 nm (515 nm to 483 nm) was observed indicating enhanced exposure of solvent accessible hydrophobic clusters. In agreement with previous finding, the maximum ANS-FI of protein was observed at pH 1.0 [34] **.** However, it raised a question regarding the location of MG-state (at pH 1.5 or pH 1.0) as far-UV CD data clearly showed that the secondary structure of protein at pH 1.0 is too less for a MG-state. Therefore further experiments were conducted to resolve this problem.

It is reported earlier that ANS exhibit great possibility of interacting with protein molecules not only through hydrophobic but also *via* electrostatic interactions [48], [49]. Therefore, we performed ITC experiments in order to explore the dominating interactive forces between ANS molecules and chymopapain under several conditions. The ANS binding parameters obtained from ITC results in this study are comparable to those reported for ANS binding to salt induced MG-state of cytochrome c by Kishore et al. [48]. At pH 7.4, we did not observe noticeable binding of ANS molecules with protein which was also in agreement with extrinsic spectroscopic results. The thermodynamic parameters obtained from the binding isotherms at pH 1.5 and pH 1.0 revealed that at high affinity binding site, more ANS molecules are associated with protein at pH 1.5 (10.5±0.3) than at pH 1.0 (8.9±0.2). Besides, the fact that binding of ANS occurred mainly at the expense of large entropy change indicates that hydrophobic interactions were predominant in the binding process at high affinity binding site [38], [50]. In other words, the hydrophobic clusters of protein were more accessible to ANS at pH 1.5. In contrast, the low affinity binding site of protein seemed to be occupied with more number of ANS molecules at pH 1.0 (22.1±1.1) than at pH 1.5 (15.2±2.0). Also, the binding of ANS to this site was derived by a considerable change in enthalpy in addition to change in entropy suggesting the involvement of electrostatic interactions. Thus a considerably larger number of electrostatically bound (along with relatively fewer hydrophobically interacted) ANS molecules may be responsible for maximum ANS-FI at pH 1.0 as observed in extrinsic fluorescence results. This is further supported by the fact that in case of bovie serum albumin, an increase in net positive charge on protein below pH 4.0 led to increase in relative fluorescence intensity and thus the number of bound ANS moleculs from 5 at pH 4.0–11.0 to ∼12 at pH 1.0 [37]. One might argue that electrostatic interactions do not necessarily contribute to the fluorescence intensity of the ANS-protein complex; however it has been reported that ANS fluorescence production depends exclusively on absence of water molecules quenching the fluorescence and not on the polar *vs.* nonpolar nature of ANS’s immediate surrounding [37]. In our case, the protein molecule is remarkably less expanded at pH 1.0 compared to denatured state as observed in DLS experiments, therefore it is very likely that immediate surroundings of some of the ANS molecules that are electrostatically bound to chymopapain are not fully accessible to water molecules for fluorescence quenching, thereby leading to an enhancement in overall fluorescence intensity. Nonetheless, the ITC data showed that non-specific interactions may, sometime, give false results and that hydrophobic clusters of chymopapain were actually more intact at pH 1.5 than at pH 1.0. Obviously the difference in the number of ANS molecules bound hydrophobically at pH 1.5 and pH 1.0 is not very large. However when considered together with the difference in the secondary structural contents and the ratio [θ]_208_/[θ]_222_ of the protein under the same conditions, the possibility of existence of MG-state appear to be far greater at pH 1.5 than at pH 1.0. Also, it should be noted that the increment in ANS-FI at pH 1.5 (∼30 times more as compared to the native state) is far greater than that observed for the MG-state of ficin (∼7 times) and papain (∼12 times) and almost similar to that of stem bromelain (∼27 times) [9], [15], [31] all of which are closely related cysteine proteases. Besides, the observed blue shift is also comparable to that observed for the MG-state of these and other proteins [17]. All these considerations are adequate to support the facts that state at pH 1.5 is a molten globule.

We also attempted to explore as to why hydrophobic interactions (in addition to electrostatic forces) were involved to such an appreciable extent in ANS-chymopapain binding at pH 1.0. It has been reported earlier that chymopapain is a kinetically stable protein [51]. This characteristic was also reflected by acid-induced unfolding pathway of protein where conformational transitions became prominent under extreme acidic pH. Such a remarkable structural stability can be attributed to presence of extremely stable hydrophobic core. Chymopapain is a protein with about 33% non polar amino acids which impart hydrophobic character to the polypeptide. Besides, aliphatic side chains of nearly 13% basic amino acids may also be contributing towards hydrophobicity of this protein [52]. Such a stable protein is less likely to undergo complete unfolding by acidification. To confirm this fact, we determined the hydrodynamic radii of chymopapain at different pH through DLS method. The analysis of DLS data revealed marked difference in *R*_h_ at pH 1.0 (3.6 nm) and for 6M GuHCl denatured state (5.1 nm). This suggests that although with unordered structure, the protein conformation at pH 1.0 was not much extended and might have retained some hydrophobic patches that interacted with ANS molecules.

Conclusively, we propose that the intermediate state of chymopapain obtained at pH 1.5, having substantial amount of secondary structure, non-rigid tertiary contacts, increased hydrodynamic radii and maximally exposed hydrophobic clusters can be suitably referred to molten globule state. Furthermore, it was revealed that in case of chymopapain, maximum ANS fluorescence alone may not be the criteria for affirming the MG-state. The scheme in Fig. 9 represents the acid-induced unfolding pathway of chymopapain along with the proposed mode of interaction of ANS molecules at different pH as well as denatured condition. As can be seen, the protein offers more (high affinity) binding sites for hydrophobic interactions to the ANS molecules at pH 1.5. On the other hand at pH 1.0, lesser ANS molecules were bound through hydrophobic forces while electrostatic interactions (low affinity binding site) outweigh the overall mode of interaction.
